# Effects of acid antisecretory drugs on mucus barrier of the rat against 5-fluorouracil-induced gastrointestinal mucositis

**DOI:** 10.1080/00365520701811693

**Published:** 2008-04-14

**Authors:** Yoichi Saegusa, Takafumi Ichikawa, Tomohisa Iwai, Yukinobu Goso, Tomoaki Ikezawa, Motoko Nakano, Nobuaki Shikama, Katsunori Saigenji, Kazuhiko Ishihara

**Affiliations:** ^1^Department of Internal Medicine, Kitasato University Graduate School of Medicine, Sagamihara, Kanagawa, Japan; ^2^Department of Biochemistry, Kitasato University Graduate School of Medicine, Sagamihara, Kanagawa, Japan; ^3^Department of Regulation Biochemistry, Kitasato University Graduate School of Medical Sciences, Sagamihara, Kanagawa, Japan; ^4^Tokushima Research Center, Taiho Pharmaceutical Co. Ltd., Tokushima, Japan

**Keywords:** Cancer chemotherapy, 5-fluorouracil, gastrointestinal mucositis, proton-pump inhibitor, rat

## Abstract

**Objective:**

Acid antisecretory agents are used for the prophylaxis of cancer chemotherapy (CT)-induced gastrointestinal (GI) mucositis. Although these drugs seem to be clinically beneficial, data on their effects on the GI mucosal defense during CT treatment are scant. The objective of this study was to compare the effects of omeprazole, lansoprazole, and lafutidine on mucin, a major mucus component, during 5-fluorouracil (5-FU) treatment, as a CT regimen.

**Material and methods:**

Rats, weighing approximately 230 g, were divided into five groups. The control group was administered 0.5% carboxymethylcellulose orally once daily for 5 days. The second, third, fourth, and fifth groups were treated with 5-FU (50 mg/kg), 5-FU plus omeprazole (10 mg/kg), 5-FU plus lansoprazole (10 mg/kg), and 5-FU plus lafutidine (30 mg/kg) in the same way, respectively. The rats were sacrificed on the sixth day, and their stomachs and small intestines were removed. Using anti-mucin monoclonal antibodies, we compared the immunoreactivity in different areas of the rats' GI tracts as well as the mucin content.

**Results:**

Body-weight decreased in rats in the 5-FU group. Lafutidine, but neither omeprazole nor lansoprazole, inhibited the 5-FU-induced weight loss. Mucosal damage and reduced mucin content in stomach and small intestine were observed in rats receiving 5-FU alone. In the stomach, all antisecretory drugs caused the protective effects against 5-FU-induced mucosal injury and alleviation of the decreased mucin accumulation. In the jejunum and ileum, lafutidine, but neither omeprazole nor lansoprazole, ameliorated the 5-FU-induced mucosal damage and decreased mucin accumulation.

**Conclusion:**

Lafutidine could offer the possibility of more effective prevention of CT-induced mucositis through the activation of GI mucus cells.

## Introduction

In 2004, the Mucositis Study Group of the Multinational Association of Supportive Care in Cancer (MASCC) and the International Society for Oral Oncology (ISOO) published the clinical practice guidelines for the prevention and treatment of cancer chemotherapy (CT)-induced gastrointestinal (GI) mucositis [1,2]. The updated guidelines recommend either ranitidine or omeprazole for the prophylaxis of epigastric pain after treatment with cyclophosphamide, methotrexate, and 5-fluorouracil (5-FU) or treatment with 5-FU with or without folinic acid [3]. The potential utility of omeprazole in the prevention of CT-induced gastroduodenal injury has been clearly demonstrated by the randomized trials in Europe [4,5]. In Japan, anti-ulcer drugs such as lansoprazole and lafutidine are given prophylactically to patients during CT treatment in the absence of randomized controlled trials. Although these drugs seem to be clinically beneficial in reducing gastric acid secretion [6,7], data on their effects on the GI mucosal defense mechanisms during CT treatment are scant.

Mucin, a major component of mucus, is considered to be one of the principal factors in the physiological defense of the GI mucosa. In our previous studies, we have reported quantitative and qualitative changes in GI mucin in experimental animals treated with various drugs including 5-FU, and demonstrated its importance in the GI mucosal barrier [8–11]. We have also established several monoclonal antibodies (mAbs) that react with mucin synthesized and secreted from specific mucus-producing cells of the rat GI mucosa [12,13].

The first objective of the present study was to compare the efficacy of omeprazole, lansoprazole, and lafutidine against 5-FU-induced rat GI mucosal injury. Secondly, we sought to evaluate their effects on mucin accumulation in different areas of the GI tract.

## Material and methods

### Animals and drug treatment

Seven-week-old male Wistar rats purchased from CLEA-Japan (Tokyo, Japan) were used in this study. These animals were housed in our animal care facility for 1–2 weeks while body-weight stabilized. The animals were housed in individual cages with raised mesh bottoms and in a temperature- and humidity-controlled environment with a 12-h dark–light cycle (1800–0600 h dark cycle). At the beginning of the experimental period, the animals were weighed after fasting for 24 h. During the below-mentioned treatment, rats were given food and water ad *libitum*. After 24 h of food deprivation following final administration of drugs, the animals were again weighed, sacrificed, and their stomachs, proximal and distal small intestines (corresponding to the jejunum and ileum, respectively) were removed. The present study was conducted according to the guidelines of the Animal Laboratory Center of Kitasato University School of Medicine.

5-FU was administered orally by gavage (50 mg/kg) once daily for 5 days. Anti-ulcer drugs used were omeprazole, lansoprazole (Sigma-Aldrich Corp., St. Louis, Mo., USA), and lafutidine (Taiho Pharm. Co. Ltd., Tokyo, Japan). All drugs were suspended in 0.5% carboxymethylcellulose (CMC) solution and prepared immediately before use. Each anti-ulcer drug (omeprazole 10 mg/kg; lansoprazole 10 mg/kg; lafutidine 30 mg/kg) was given orally 30 min before the respective 5-FU administration. Control animals received 0.5% CMC instead of 5-FU and anti-ulcer drugs.

### Histological examination

Specimens of each tissue were immediately fixed for 3 h in freshly prepared Carnoy's solution following the method described elsewhere [14]. After fixation, the materials were dehydrated through ethanol, cleared in xylene and embedded in paraffin. From these specimens, 3-μm paraffin sections were prepared for immunostaining with anti-mucin mAbs. Immunohistochemical staining was done using the avidin-biotin peroxidase method and an LSAB2 Kit (Dako, Carpinteria, Calif., USA). Briefly, endogenous peroxidase activity was blocked with 0.3% H_2_O_2_, and then the tissue was sequentially incubated with 10% (v/v) normal swine serum, the anti-mucin mAb (RGM21, RGM26, PGM34), biotinylated anti-mouse immunoglobulins, streptavidin horseradish peroxidase (HRP), and 0.02% 3,3-diaminobenzidine in 50 mM Tris-HCl, pH 7.6, containing 0.005% H_2_O_2_. The counterstaining was done with hematoxylin. The immunohistochemical reactivity of each of the mAbs was observed using an optical microscope. As previously described [11,12], the immunohistochemical reactivities of RGM21 and RGM26 were located in the surface epithelial mucus cells of the rat corpus and antral mucosa, respectively. Regarding PGM34, it was recently shown that the epitope of this mAb was a specific sulfated oligosaccharide of the mucin molecule. This mAb stains all the goblet cells of rat small intestine [13].

### Biochemical examination

Specimens from each tissue were lyophilized and powdered for extraction of mucin by the previously described method [8]. Each sample was suspended in 50 mM Tris-HCl, pH 7.2, containing 2% Triton X-100 (Triton-Tris buffer), homogenized and then incubated at 37°C for 1 h. After centrifugation at 8000 *g* for 30 min at 4°C, the supernatant was collected and an aliquot was applied to a Bio-Gel A-1.5 m column, and eluted with the Triton-Tris buffer. The void volume fraction (Fr-1) monitored by hexose measurement was collected as mucin. Hexose content in this fraction was measured by the phenol-sulfuric acid method using galactose as the standard. Mucin content (Fr-1 hexose value) was expressed as micrograms of hexose per tissue.

### Statistical analysis

The difference in the mean values among the groups was analyzed by one-way ANOVA with Scheffe's test; a *p*-value of less than 0.05 was considered to indicate statistical significance.

## Results

### Body-weight change

The changes in the body-weight of the rats in each experimental group are recorded in Table I. During a 6-day period, a body-weight gain was seen in rats of the control group, whereas a weight loss was found in the animals given 5-FU orally at a dose of 50 mg/kg once daily for 5 consecutive days. There were virtually no changes in the weights of rats administered 5-FU with either omeprazole or lansoprazole. There was a slight tendency toward an increase in the lafutidine plus 5-FU group, indicating that lafutidine inhibits 5-FU-induced body weight loss.

**Table I:** Weight changes in rats before and after the treatments.

		Body Weight	
		
	*n*	Before	After
Control	9	229.8 (±6.2)	256.4 (±6.1)*
5-FU	8	231.0 (±8.7)	204.2 (±10.2)*
Ome+5-FU	7	227.3 (±17.5)	220.7 (±11.8)*
Lan+5-FU	6	226.2 (±5.3)	220.2 (±4.8)*
Laf+5-FU	9	238.1 (±6.2)	245.0 (±11.3)*

Mean (±SD)

* *p*<0.05; 5-FU=5-fluorouracil; Ome=omeprazole; Lan=lansoprazole; Laf=lafutidine.

### Changes in immunoreactivity and mucin content of the gastric mucosa

**Figure 1:**
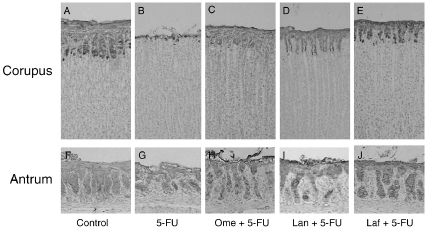
Immunostaining of the gastric corpus (A–E) and antral (F–J) mucosae with anti-mucin monoclonal antibodies. Gastric tissues were obtained from control rats (A, F), rats treated with 5-fluorouracil (5-FU) alone (B, G), rats treated with omeprazole (Ome+ 5-FU (C, H), rats treated with lansoprazole (Lan)+5-FU (D, I), and rats treated with lafutidine (Laf)+5-FU (E, J). Notice that surface epithelial mucus cells in the corpus show positive staining with RGM21, and those in the antrum show positive staining with RGM26. Original magnification ×25.

Figure 1 shows the morphological changes in corpus and antral mucosae after the treatments. In the control rat, gastric surface epithelial mucus cells were strongly stained with anti-mucin mAbs (RGM21 and RGM26, Figure 1A and F, respectively). Treatment with 5-FU caused gastric mucosal damage restricted to the superficial epithelium [11]. This was characterized by significant decreases in the RGM21- and RGM26-immunoreactivities in the corpus and antrum, respectively, when compared with the individual control (Figure 1B, G). In contrast, significant observable damage could rarely be found in the gastric mucosae of the animals with the combined application of 5-FU and omeprazole, lansoprazole, or lafutidine (Figure 1C–E, H–J).

**Figure 2:**
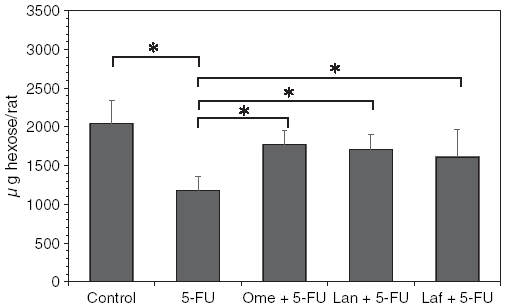
Influence of acid antisecretory agents on the gastric corpus mucin accumulation in the 5-FU-induced gastric mucosal damage. Fr-1 hexose values corresponding to mucin content are expressed as micrograms of hexose per rat and represent means ± SD. Abbreviations: 5-FU = 5-fluorouracil; Ome = omeprazole; Lan = lansoprazole; Laf = lafutidine. *n* = 6–9 (each group); *p < 0.05.

Figure 2 shows the effect of omeprazole, lansoprazole, or lafutidine treatment on the corpus mucin content in the 5-FU-induced gastric mucosal damage expressed as the Fr-1 hexose value. In the corpus of the rats treated with 5-FU, the mucin content was significantly decreased to 57.6% of the control. The 5-FU-induced mucin reduction was inhibited by the combination treatment of omeprazole, lansoprazole, or lafutidine.

### Changes in immunoreactivity and mucin content of the small-intestinal mucosa

**Figure 3:**
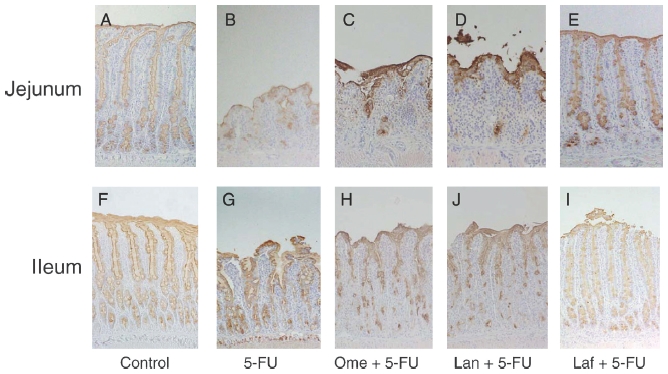
Immunostaining of the rat jejunal (A–E) and ileal (F–J) mucosae with anti-mucin monoclonal antibody PGM34. Small-bowel tissues were obtained from control rats (A, F), rats treated with 5-fluorouracil (5-FU) alone (B, G), rats treated with omeprazole (Ome)+5-FU (C, H), rats treated with lansoprazole (Lan)+5-FU (D, I), and rats treated with lafutidine (Laf)+5-FU (E, J). Notice that goblet cells in the jejunum and ileum show positive staining with PGM34. Original magnification ×25.

Figure 3 shows the morphological changes in the small-intestinal mucosa after treatments. In the control rats, immunohistochemical reactivity for PGM34 could be detected in the goblet cells, as well as the surface mucus gel layer, in the jejunum and ileum (Figure 3A, F). As shown in Figure 3B and G, 5-FU treatment caused a marked decrease in villus height and a remarkable reduction in the number of PGM34-positive goblet cells. In the animals treated with a combination of 5-FU and lafutidine, significant observable damage could rarely be found in the sections of the jejunal or ileal mucosa (Figure 3E, J), whereas neither omeprazole (Figure 3C, H) nor lansoprazole (Figure 3D, I) was shown to prevent the 5-FU-induced intestinal mucosal damage.

**Figure 4:**
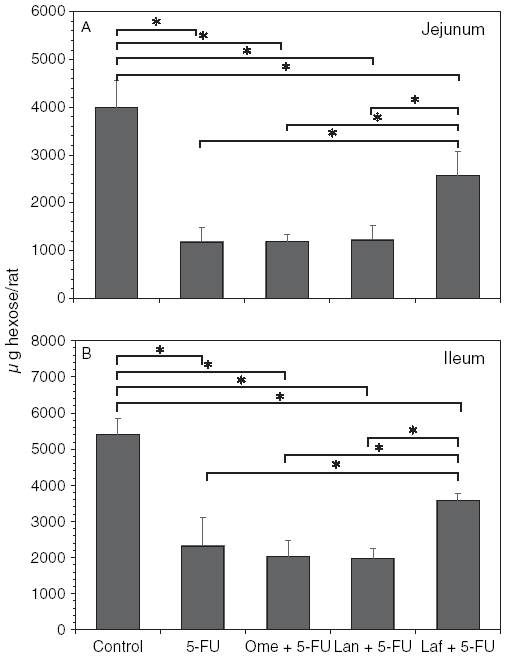
Influence of acid antisecretory agents on the jejunal (A) and ileal (B) mucin accumulation in the 5-FU-induced small-bowel mucosal damage. Fr-1 hexose values corresponding to mucin content are expressed as micrograms of hexose per rat and represent means±SD. Abbreviations: 5-FU = 5-fluorouracil; Ome = omeprazole; Lan = lansoprazole; Laf = lafutidine. *n* = 6–9 (each group); *p<0.05.

Figure 4 shows the comparison of the effects of the anti-ulcer drugs on the small-intestinal mucin contents in the 5-FU-induced mucosal damage. A decrease in the mucin content of the jejunum and ileum was observed after treatment with 5-FU (29.6% and 42.9% of the control mucin content, respectively). Lafutidine pretreatment significantly inhibited the 5-FU-induced mucin reduction in the jejunum and ileum mucin (75.8% and 66.1% of the control mucin, respectively), whereas no significant change could be detected in the mucin content in the small intestine by the 5-FU treatment with either omeprazole or lansoprazole.

## Discussion

Using the original anti-mucin mAbs RGM21 and RGM26, we demonstrated the protective effects of three anti-ulcer drugs, omeprazole, lansoprazole, and lafutidine, against 5-FU-induced gastric mucosal injury of the rat. From the randomized controlled studies, Sartori et al. [4,5] documented that the strong and prolonged suppression of gastric acid secretion by omeprazole was effective in preventing and reducing CT-induced gastroduodenal mucosal injury, suggesting an important prophylactic role of the inhibition of acid secretion. Both lansoprazole and lafutidine possess a potent and long-lasting gastric antisecretory effect in humans [6,7]. In the rat models, each drug at the dose used in this study has been shown sufficiently to decrease both the basal and the stimulated acid secretion [15–18]. Our results strongly support the clinical studies showing that the acid-inhibitory drugs such as proton-pump inhibitors (PPIs) and H_2_-blockers are effective in reducing the frequency of gastric mucosal injury and upper GI symptoms caused by CT treatment [4,5,19].

In the stomach, mucin is a key element in protecting the gastric epithelium against various irritants [8,10,20]. Changes in gastric mucin content have been shown to occur in association with the oral administration of certain chemical agents including aspirin and 5-FU [8,10,11]. In this study, a significant decrease in the mucin content of corpus mucosa was noted after oral administration of 5-FU at a dose of 50 mg/kg once daily for 5 consecutive days. Our most notable finding was that the mucin content did not decrease in animals given each of the anti-ulcer drugs used in this study. Accumulation of mucin in the gastric mucosa is closely related to mucosal protective capability [8–10]. We have already reported that lafutidine, independent of its histamine H_2_-receptor antagonistic property, exerts a stimulant activity in the mucin accumulation and the protective effect against necrotizing-agent-induced gastric mucosal damage in the rat [9]. Moreover, our recent study showed that lafutidine, given at clinical dosages, not only inhibits acid secretion but also strengthens the mucus barrier of the human gastric mucosa [21]. The preventive effects of three anti-ulcer drugs against the 5-FU-induced gastric mucosal injury might also be associated with the non-acid inhibitory mechanism including mucosal defensive factors.

As we have previously demonstrated [11], oral administration of 5-FU caused the remarkable decreases in both the number of mucus cells and the mucin content in the rat small intestine, especially in the jejunum. Here we report on a preventive effect of lafutidine on 5-FU-induced alteration in the rat intestinal mucus. Although the protective property of intestinal mucin has received limited attention compared with gastric mucin [22], our results suggest that lafutidine may be extremely useful in reducing CT-induced GI mucosal damage. Recent studies have documented the prophylactic effect of this drug on indomethacin-induced small-intestinal ulcers in rats [23,24]. Moreover, our study showed that lafutidine had an effect on body-weight loss in the animals treated with 5-FU. Although accurate measurement of either food intake or fecal output was not done in our investigation, lafutidine appeared to prevent the 5-FU-induced hypophagia and ingestion. These findings should be confirmed in future large randomized controlled clinical trials of lafutidine during CT treatment.

Anti-neoplastic drugs may cause severe damage to normal cells in organs with a high cellular turnover. Because gastric and intestinal epithelia have a high growth fraction [25], the potential risk of CT-induced injury is high. Therefore, it is possible that reduction of CT-induced GI injury may be related to a reduction in the growth-inhibitory ability of anti-cancer agents. However, to our knowledge, there are no previous reports to show that the anti-ulcer drugs used in this study will lead to a decreased anti-tumor efficacy in cancer CT treatment. Dilloway & Lant [26] reported that a non-imidazole-based H_2_-blocker did not cause any significant effect on 5-FU pharmacokinetics in the rat and monkey, suggesting that lafutidine could not reduce the 5-FU blood levels. In the preliminary study using a Yoshida sarcoma-bearing rat model, lafutidine had no influence on the anti-tumor activity of TS-1 (30 mg/kg p.o.), a prodrug of 5-FU (data not shown). Our previous report showed that lafutidine directly stimulated the mucin production in the rat mucus cells [27,28]. Thus, the preventive effect of lafutidine against 5-FU-induced intestinal damage may be attributed to the increased production of mucin by the goblet cells that remained alive after 5-FU treatment.

Omeprazole and lansoprazole have been shown to ameliorate intestinal mucosal damage induced by indomethacin or ischemia-reperfusion in rats, via the action being dependent on their anti-inflammatory and anti-oxidative responses [29–31]. In this study omeprazole and lansoprazole failed to alleviate the changes in both the morphological defects and mucin contents in intestinal mucosae of rats treated with 5-FU. We previously found that omeprazole had no effect on mucin biosynthesis in the rat gastric mucosa [27]. These findings suggest that the omeprazole and lansoprazole utilized in this study could not promote the goblet mucus cell function. Although further studies are needed to clarify the detailed mechanism for 5-FU-induced intestinal injury, the activation of the goblet cells, if appropriately manipulated, might lead to more effective prevention of 5-FU-induced GI mucositis.

To summarize, we present two important research findings. First, oral administration of omeprazole, lansoprazole, and lafutidine caused the protective effects against 5-FU-induced gastric mucosal injury through alleviation of the decreased mucin accumulation in the rat stomach. Second, lafutidine ameliorated the intestinal mucosal damage and the decreased body-weight gain induced by 5-FU treatment, raising the possibility of a more effective prevention of CT-induced GI mucositis.
